# Mutational Analysis of Hedgehog Signaling Pathway Genes in Human Malignant Mesothelioma

**DOI:** 10.1371/journal.pone.0066685

**Published:** 2013-06-24

**Authors:** Chuan Bian Lim, Cecilia M. Prêle, Hui Min Cheah, Yuen Yee Cheng, Sonja Klebe, Glen Reid, D. Neil Watkins, Svetlana Baltic, Philip J. Thompson, Steven E. Mutsaers

**Affiliations:** 1 Lung Institute of Western Australia and Centre for Asthma, Allergy and Respiratory Research, Department of Medicine, School of Medicine and Pharmacology, University of Western Australia, Crawley, WA, Australia; 2 Centre for Cell Therapy and Regenerative Medicine, School of Medicine and Pharmacology and Western Australian Institute for Medical Research, University of Western Australia, Crawley, WA, Australia; 3 Asbestos Diseases Research Institute (ADRI), University of Sydney, Sydney, NSW, Australia; 4 Department of Anatomical Pathology, SA Pathology and Flinders University, Flinders Medical Centre, Adelaide, Australia; 5 Centre for Cancer Research, Monash Institute for Medical Research, Monash University, Melbourne, Victoria, Australia; University of South Florida College of Medicine, United States of America

## Abstract

**Background:**

The Hedgehog (HH) signaling pathway is critical for embryonic development and adult homeostasis. Recent studies have identified regulatory roles for this pathway in certain cancers with mutations in the HH pathway genes. The extent to which mutations of the HH pathway genes are involved in the pathogenesis of malignant mesothelioma (MMe) is unknown.

**Methodology/Principal Findings:**

Real-time PCR analysis of HH pathway genes *PTCH1*, *GLI1* and *GLI2* were performed on 7 human MMe cell lines. Exon sequencing of 13 HH pathway genes was also performed in cell lines and human MMe tumors. *In silico* programs were used to predict the likelihood that an amino-acid substitution would have a functional effect. *GLI1*, *GLI2* and *PTCH1* were highly expressed in MMe cells, indicative of active HH signaling. *PTCH1*, *SMO* and *SUFU* mutations were found in 2 of 11 MMe cell lines examined. A non-synonymous missense *SUFU* mutation (p.T411M) was identified in LO68 cells. *In silico* characterization of the *SUFU* mutant suggested that the p.T411M mutation might alter protein function. However, we were unable to demonstrate any functional effect of this mutation on Gli activity. Deletion of exons of the *PTCH1* gene was found in JU77 cells, resulting in loss of one of two extracellular loops implicated in HH ligand binding and the intracellular C-terminal domain. A 3-bp insertion (69_70insCTG) in *SMO*, predicting an additional leucine residue in the signal peptide segment of SMO protein was also identified in LO68 cells and a MMe tumour.

**Conclusions/Significance:**

We identified the first novel mutations in *PTCH1*, *SUFU* and *SMO* associated with MMe. Although HH pathway mutations are relatively rare in MMe, these data suggest a possible role for dysfunctional HH pathway in the pathogenesis of a subgroup of MMe and help rationalize the exploration of HH pathway inhibitors for MMe therapy.

## Introduction

Malignant mesothelioma (MMe) is an aggressive, incurable cancer that arises from mesothelial cells that line the serosal cavities of the pleura, peritoneum, pericardium and tunica vaginalis testis. Occupational asbestos exposure is the main risk factor for MMe, accounting for 80% of the cases in men and 40% of the cases in women [1]. The global incidence of MMe has been on the rise since the 1960s and is projected to increase until at least 2050 due to the widespread use of asbestos during the past decades [2]–[5]. MMe is highly refractory to current treatment modalities including chemotherapy, radiotherapy and surgery, with a short median survival time of less than 12 months after first diagnosis [6]. There is no doubt that successful treatment will require a paradigm shift in how MMe is viewed as a disease. Current research is focusing on the molecular mechanisms underlying MMe development and growth to identify new targets for therapeutic intervention [7].

**Figure 1:**
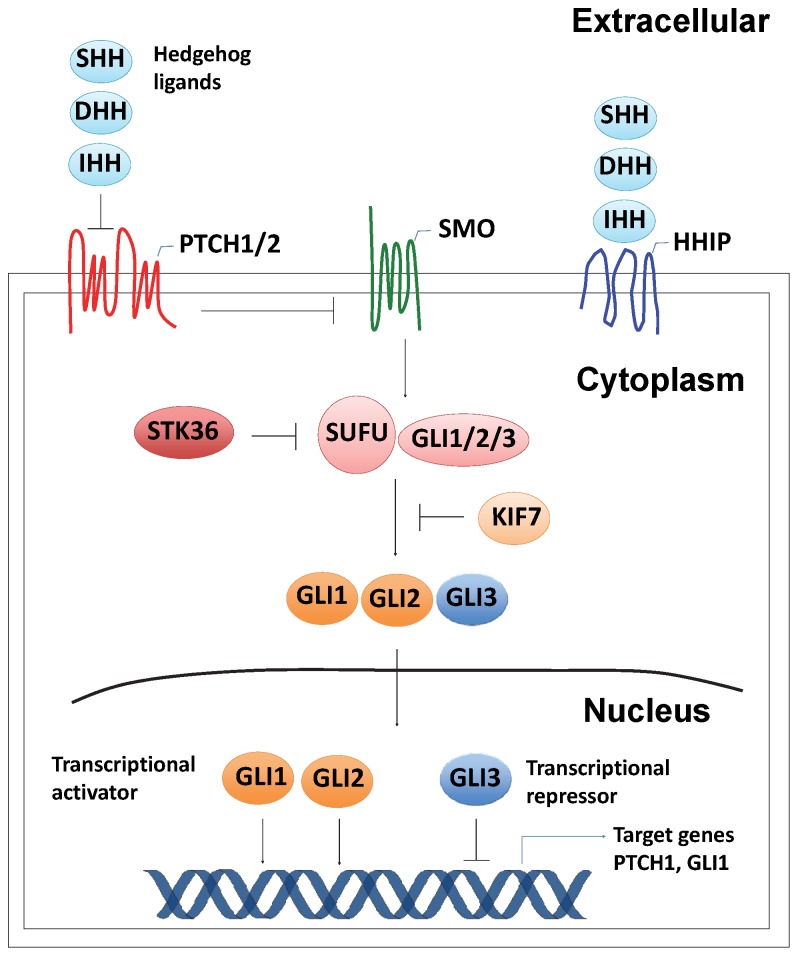
Hedgehog signaling pathway. HH ligands (SHH, DHH or IHH) bind to the PTCH receptors (PTCH1 and PTCH2) and relieve the inhibition of SMO. HHIP, a negative inhibitor of HH signaling, can compete with PTCH receptors to bind HH ligand, resulting in the attenuation of HH signaling. SMO then transduces signals through the cytoplasmic SUFU-GLI complex, resulting in the activation and nuclear translocation of the downstream GLI transcription factors (GLI1-3). STK36 further promotes activation and nuclear accumulation of GLI by antagonizing SUFU. KIF7 regulates GLI-mediated transcription both positively and negatively through physical interaction with GLI and regulating the stability and degradation of GLI proteins.

The Hedgehog (HH) signaling pathway regulates critical aspects of embryonic development as well as adult tissue homeostasis and stem cell maintenance [8]–[10]. Recently, the HH pathway has been implicated as a major contributor to the growth and maintenance of a variety of human cancers [11]. HH acts as a ligand for the Patched (PTCH) receptor proteins, PTCH1 and PTCH2 [12]. In the absence of the HH ligand (Sonic HH (SHH), Desert HH (DHH) and Indian HH (IHH)), PTCH interaction with Smoothened (SMO) inhibits SMO function [13]. Upon ligand binding, PTCH-mediated repression of SMO is relieved and SMO transduces the signal to a SUFU-GLI complex residing in the cytoplasm, resulting in the release and activation of GLI transcription factors [14]. SUFU is the main repressor of the mammalian HH signaling pathway by sequestering GLI transcription factors in the cytoplasm and nucleus [15]. This repressor is negatively regulated by serine/threonine kinase 36 (STK36), which in turn promotes activation and nuclear accumulation of GLI [16]. In addition to SUFU, the transmembrane HH-interacting protein (HHIP) was identified as another negative regulator of the HH pathway that acts by sequestering all three HH homologs with similar affinity to that of PTCH1 protein [17]. Recently, KIF7 was identified by sequence comparison as a GLI-interacting protein [18]. KIF7 physically binds to GLI, regulating their stability and degradation and controlled GLI-mediated transcription [19]. There are 3 GLI proteins in vertebrates; GLI1, GLI2 and GLI3, which are capable of either transcriptional activation or repression of cell type-specific HH pathway target genes [20]. A schematic diagram depicting the Hedgehog signaling pathway is shown in Figure 1.

Mutations in *PTCH1* have been found in familial basal cell carcinoma (BCC) and medulloblastoma (MB) [21], [22]. It was later discovered that sporadic BCCs harbored somatic *PTCH1* mutations [23]. *PTCH1* mutations have also been found in other sporadic tumors, such as MB [24], skin trichoepitheliomas [25], esophageal squamous cell carcinomas [26], skin squamous cell carcinomas [27], meningiomas [24], breast carcinomas [24] and bladder carcinomas [28], as well as in odontogenic keratocysts [24], [27], [29]–[31]. In addition to primary tumors, missense mutations in *PTCH1* have been identified in several oral squamous cell carcinoma and colon carcinoma cell lines [24], [32].

Clearly mutations in *PTCH1* do not account for all cases of familial as well as sporadic BCCs and MBs. Indeed, mutations in several genes in the HH pathway have been found in familial and sporadic BCCs and MBs. In a subset of patients with sporadic BCC, mutations in *SMO* have been described [33]–[37] while *PTCH2* mutations were detected in some cases of sporadic BCCs and MBs [38]. Unlike PTCH1, PTCH2 is overexpressed in both familial and sporadic BCCs [39] and MBs [40], suggesting that PTCH2 is a direct gene target of HH signaling and that PTCH2 may be negatively regulated by PTCH1 [39]. Loss of *PTCH2* has been reported to contribute to enhanced tumorigenesis in PTCH1 haploinsufficient mice [41].

Mutations in *SUFU* were detected in sporadic BCCs and seemed to predispose to MB [33], [42]. Mutations in *SHH* have also been identified in a number of cancer types, including BCC [43], MB [43], breast carcinoma [43], glioblastoma [44] and lung cancer [45]. Oro and coworkers detected an identical somatic mutation in the putative zinc hydrolase site of SHH that caused a histidine to tyrosine substitution at codon 133 of SHH. Notably, this mutation occurred in a sporadic BCC, a MB and a breast carcinoma, hinting at the possible involvement of this nonsynonymous substitution in driving malignancies in these tumor types [43].

To our knowledge, mutations in HH pathway genes have not yet been reported in MMe. In this study, we report identification of a novel multi-exonic deletion in *PTCH1* and an insertion mutation in *SMO* in human MMe, identified via direct sequencing of genes encoding HH signaling pathway proteins in a panel of 7 MMe cell lines. We also identified a point mutation close to the Gli-binding domain of *SUFU* that led to the replacement of threonine for methionine at amino acid position 411. However, Gli1 luciferase reporter assays revealed that this mutation failed to disrupt the inhibitory function of SUFU. We also genotyped a collection of primary tumors for the presence of these mutations and found one tumor sample that carried the *SMO* insertion mutation. In addition, we also detected a number of previously reported single nucleotide polymorphisms (SNPs) in the course of our screen. Six of these sequence variants were predicted to have substantial impact upon the function of the encoded protein.

## Materials and Methods

### Cell Lines and Culture Conditions

The human MMe cell lines MSTO-211H (CRL-2081), NCI-H28 (CRL-5820), NCI-H226 (CRL-5826), NCI-H2052 (CRL-5915) and NCI-H2452 (CRL-5946) as well as the mouse embryonic fibroblasts C3H/10T1/2 (CCL-226) were obtained from the American Type Culture Collection (ATCC). The 5 MMe cell lines JU77, LO68, NO36, ONE58 and STY51 were kind gifts from Professor Bruce W. Robinson, University of Western Australia, Australia [46] and REN cells were provided by Professor Steve Albelda, University of Pennsylvania, USA. These cell lines were cultured in high glucose Dulbecco’s modified Eagle’s medium (DMEM) supplemented with 10% fetal bovine serum, 4 mM L-glutamine, 100 units/ml penicillin and 100 µg/ml streptomycin (all from Invitrogen) in a humidified incubator with 5% CO_2_ at 37^o^C. In addition, normal pericardial mesothelial cells were obtained from patients undergoing thoracic surgery as described previously [47].

### Ethics Statement

Use of the archival tissue blocks in this retrospective study was approved by the Sydney Local Health District Human Research Ethics Committee (Concord Hospital), as part of a larger study to identify prognostic factors in malignant pleural mesothelioma. Consent for the use of these samples was waived by the ethics committee, consistent with the Human Tissue Act (1983) and the NHMRC National Statement on Ethical Conduct in Human Research (Commonwealth of Australia, 2007).

### Patients and Tumors

The MPM tumour samples used in this study (Table 1) were part of a series collected from patients who underwent extrapleural pneumonectomy at Royal Prince Alfred or Strathfield Private Hospitals (Sydney, Australia) between 1994 and 2009 [48]. The tumour content in the formalin-fixed paraffin embedded (FFPE) blocks was marked on whole sections to enable enrichment of tumour content via subsequent laser-capture microdissection.

**Table 1:** Demographic and clinical characteristics of patients with Mme.

Sample ID	Subtype	Gender	Age	Survival (months)
24	Epithelioid	M	53	9.56
31	Biphasic	M	60	5.42
32	Epithelioid	M	58	41.4
48	Epithelioid	F	48	44.16
50	Epithelioid	M	54	72.9
51	Biphasic	M	51	3.42
52	Epithelioid	M	61	8.28
59	Epithelioid	M	52	13.73
65	Epithelioid	M	64	6.93
72	Epithelioid	M	47	1.94
74	Epithelioid	M	57	8.34
77	Biphasic	M	56	2.56
78	Epithelioid	F	59	14.52
79	Epithelioid	M	56	10.32

### RNA Isolation, cDNA Synthesis and Quantitative Real-time PCR Analysis (qRT-PCR) of Gene Expression

Total RNA was extracted from cell lines using an RNeasy Mini kit (Qiagen). Two micrograms of total RNA was reverse-transcribed into cDNA using random primers (Invitrogen) and Omniscript RT kit (Qiagen). TaqMan gene expression assays (Applied Biosystems) were used for quantifying the mRNA expression levels of *GLI1*, *GLI2* and *PTCH1*. *PGK1* was included as the endogenous control. Real-time PCR was carried out on the StepOne Plus Real-Time PCR System (Applied Biosystems) in duplicates. Fold change in gene expression relative to *PGK1* was calculated using the formula as follows:
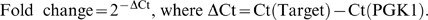



### DNA Isolation, PCR Amplification and DNA Sequence Analysis

Genomic DNA was isolated from cells using the PureLink Genomic DNA kit (Invitrogen) and from FFPE samples using the FFPE DNA minikit (Qiagen), each according to the manufacturer’s instructions. PCR primers that amplify the exons and flanking intronic sequences of 13 HH pathway genes (Table 2) were either obtained from published literature [49], [50] or designed using GenBank sequences and the Vector NTI 11.0 software. The primer sequences are listed in Table S1. PCR amplification of HH pathway genes was performed as described previously with slight modifications [51]. PCR was carried out on a iCycler thermal cycler (BioRad) in a 20 µl volume containing 10 ng genomic DNA, 1x GoTaq Flexi buffer (Promega), 1.5 mM MgCl_2_ (Promega), 0.2 mM dNTPs (Invitrogen), 0.5 µM primers (Geneworks Pty Ltd), 6% DMSO (Sigma Aldrich), 1.25 U GoTaq Flexi DNA polymerase (Promega). The optimized PCR conditions for each primer pair are listed in Table S2. PCR products were visualized on 1% agarose gels before they were sequenced at the Australian Genome Research Facility (AGRF), Perth, Western Australia, using a Big Dye Terminator v3.1 cycle sequencing kit (Applied Biosystems) and analyzed on a 3730*xl* DNA Analyzer (Applied Biosystems). Base calling and quality assessment were carried out using the Sequence Scanner v1.0 software (Applied Biosystems) while sequence assembly was carried out using the Vector NTI v11.0 software (Invitrogen). All sequence variants found were confirmed by an independent PCR and sequencing reaction to exclude PCR artifacts. Seven mesothelioma cell lines (MSTO-211H, NCI-H28, JU77, LO68, NO36, ONE58 and STY51) were originally exon sequenced with a further 4 cell lines (NCI-H226, NCI-H2052, NCI-H2452 and REN) genotyped for identified mutations.

**Table 2:** Genes sequenced in this study.

Gene Symbol	Gene name	NCBI Gene ID
*GLI1*	GLI family zinc finger 1	2735
*GLI2*	GLI family zinc finger 2	2736
*GLI3*	GLI family zinc finger 3	2737
*IHH*	indian hedgehog	3549
*PTCH1*	patched 1	5727
*SHH*	sonic hedgehog	6469
*SMO*	smoothened, frizzled family receptor	6608
*PTCH2*	patched 2	8643
*STK36*	serine/threonine kinase 36	27148
*DHH*	desert hedgehog	50846
*SUFU*	suppressor of fused homolog (Drosophila)	51684
*HHIP*	hedgehog interacting protein	64399
*KIF7*	kinesin family member 7	374654

### PCR Assay for Detection of PTCH1 Exon Deletions

To detect exon deletions of the *PTCH1* gene in MMe cell lines, we designed a PCR assay using exonic primers directed against exons 18, 19, 20, 21, 22, and 23 of *PTCH1*. The PCR condition (Protocol 1) and primer sequences are listed in Tables S2 and S3, respectively. PCR products were visualized on 4% agarose gels.

### *In silico* Characterization of Polymorphisms in Exons

The web-based programs SIFT [52] and PolyPhen2 [53] were employed to predict the potential effect of non-synonymous amino acid substitutions resulting from the genetic alterations. The default settings were used for all parameters of each program.

### Gli Luciferase Reporter Assay

C3H/10T1/2 cells were plated in triplicates on 24-well plates 24 h before transfection. Cells were cotransfected with 25 ng *pRL-TK (Promega)*, 0.1 µg 12XGLI1 luciferase reporter construct (a kind gift from Professor Rune Toftgård, Karolinska Institutet), 0.15 µg wild-type *GLI1* construct (Origene) and 0.15 µg of the appropriate *SUFU* construct (wild-type *SUFU* or *SUFU* (p.T411M) (Blue Heron Bio) using FuGene 6 transfection reagent (Roche), with a 3∶1 ratio (v/w) of FuGene 6 to DNA. Cells were harvested using the Dual-Glo Luciferase assay system (Promega), 48 h after transfection according to the manufacturer’s instruction. Luciferase activity was measured using a Wallac 1420 VICTOR2 multilabel plate reader (Perkin Elmer). All reporter assays were normalized to *Renilla luciferase activity*. All transfections were repeated in at least two independent experiments, which gave reproducible results.

### Statistical Analysis

Statistical calculations were performed using Graphpad Prism 4.03 software (Graphpad Software, Inc.). Student’s t-test was used to determine the significance of luciferase reporter assays, and p<0.05 was considered significant.

## Results

### The Canonical HH Signaling Pathway is Active in Human MMe Cell Lines

**Figure 2:**
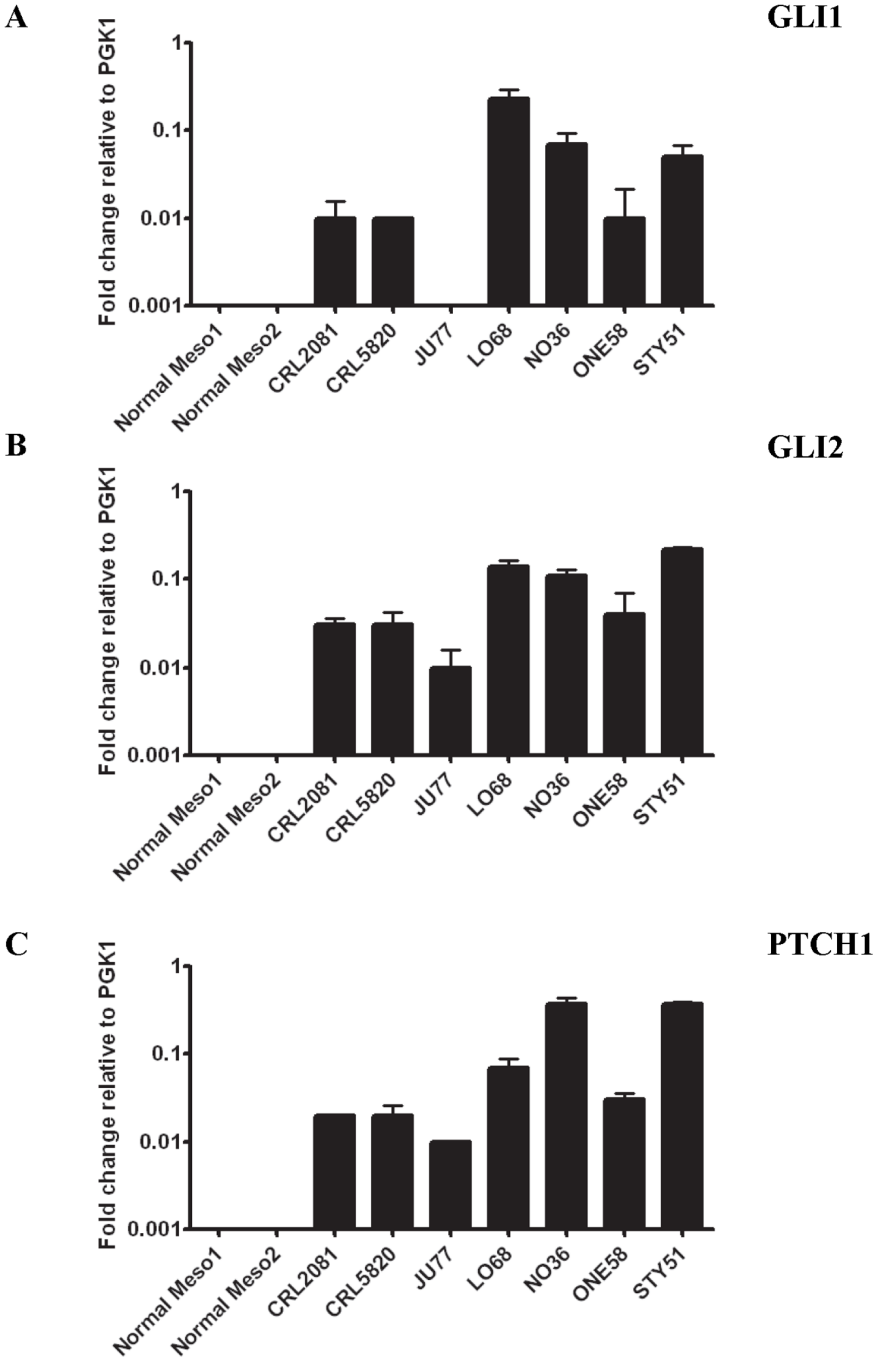
Relative expression (expressed in Δ*C*T) of the HH pathway genes. For each gene, the relative expression of mRNA was normalized to an endogenous *PGK1* control. Values represent the mean ± S.E.M. of three independent experiments each performed in duplicates. **A**, *GLI1*, **B**, *GLI2* and **C**, *PTCH1* in 7 MMe cell lines and 2 primary cultures of normal mesothelial cells.

We used qRT-PCR to demonstrate the mRNA expression of transcription factors *GLI1* and *GLI2*, activators of the canonical HH signaling pathway and a downstream HH target gene *PTCH1*, in a panel of 7 human MMe cell lines (Figure 2). In contrast, normal mesothelial cells expressed extremely low levels of *PTCH1* and *GLI2* mRNA while *GLI1* was undetectable (Figure 2). These data suggest that the components necessary for active HH signaling are expressed at significantly higher levels in all MMe cell lines analyzed, compared with controls. This is consistent with recent findings of *GLI1* and HH interacting protein (*HHIP*) being elevated 2–6-fold in MMe tissues using qRT-PCR analysis, relative to benign pleural tissue [54]. In a similar study, the mRNA and protein levels of *GLI1*, GLI2 and the signal transducer SMO were found to be overexpressed in 46 MMe tissues, as analyzed by qRT-PCR and immunohistochemistry [55].

### PTCH1, SMO and SUFU Mutations in MMe Cell Lines

**Figure 3:**
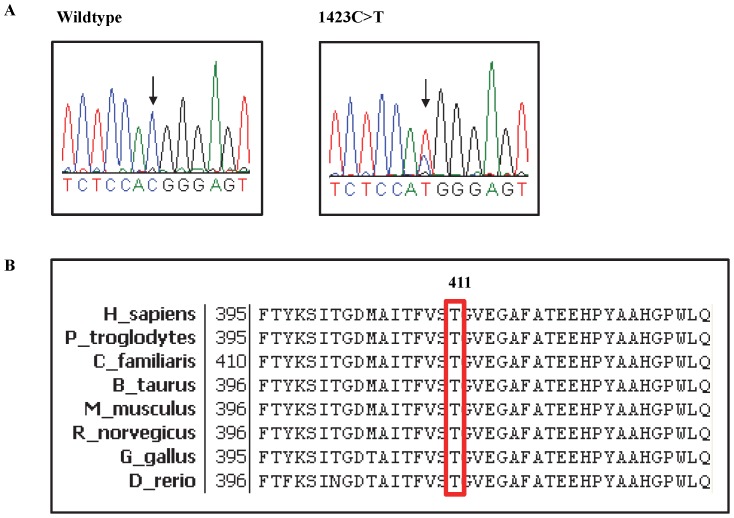
Identification of a *SUFU* ^T411M^ mutation in LO68 cell line. **A**, Electropherogram of *SUFU* gene. Left, the sequencing result of MSTO-211H cell line, showing the wildtype *SUFU* gene. Right, the sequencing result of LO68 carrying the mutant allele. **B**, Amino acid sequence alignment of SUFU from various species. Shown in the red box is the T411 residue that is evolutionarily conserved among these different species. Numbers indicate the position of amino acid residue with the start codon (methionine) as number 1.

Through *de novo* sequencing, all the exons of 13 genes encoding components of the HH signaling pathway were screened for mutations in a panel of 7 MMe cell lines. No mutation was found in the exonic regions of *SHH, DHH, IHH, PTCH1, PTCH2, HHIP, KIF7, GLI1, GLI2 and GLI3* genes. However, one non-synonymous mutation in *SUFU* was identified (Table 3). This missense mutation, which was found in LO68, involved a C>T transition at nucleotide 1232 in exon 10 of *SUFU*, which substitutes threonine for methionine at position 411 (p.T411M) (Figure 3A). This mutation was characterized by SIFT [52] and PolyPhen2 [53] and determined to be damaging. We have also cross-checked with 1000 Genomes and confirmed that this insertion is not a germline variant. In addition, as shown in Figure 3B, amino acid sequence alignment from various species revealed that Thr^411^ of SUFU was evolutionarily highly conserved.

**Table 3:** Mutations identified in MMe cell lines.

Type of alteration	Gene	Exon	Region	Nucleotidechange	Amino acid change	SIFT	PolyPhen2	Cell line
Point mutation	*SUFU*	10	Coding	1232 C>T	T411M	Damaging	Possibly damaging	LO68
Deletion	*PTCH1*	18	Coding	–	–	Unknown	Unknown	JU77
	*PTCH1*	19	Coding	–	–	Unknown	Unknown	JU77
	*PTCH1*	20	Coding	–	–	Unknown	Unknown	JU77
	*PTCH1*	21	Coding	–	–	Unknown	Unknown	JU77
	*PTCH1*	22	Coding	–	–	Unknown	Unknown	JU77
	*PTCH1*	23	Coding	–	–	Unknown	Unknown	JU77
Insertion	*SMO*	1	Coding	69_70insCTG	23L_24GinsL	Unknown	Unknown	LO68

**Figure 4:**
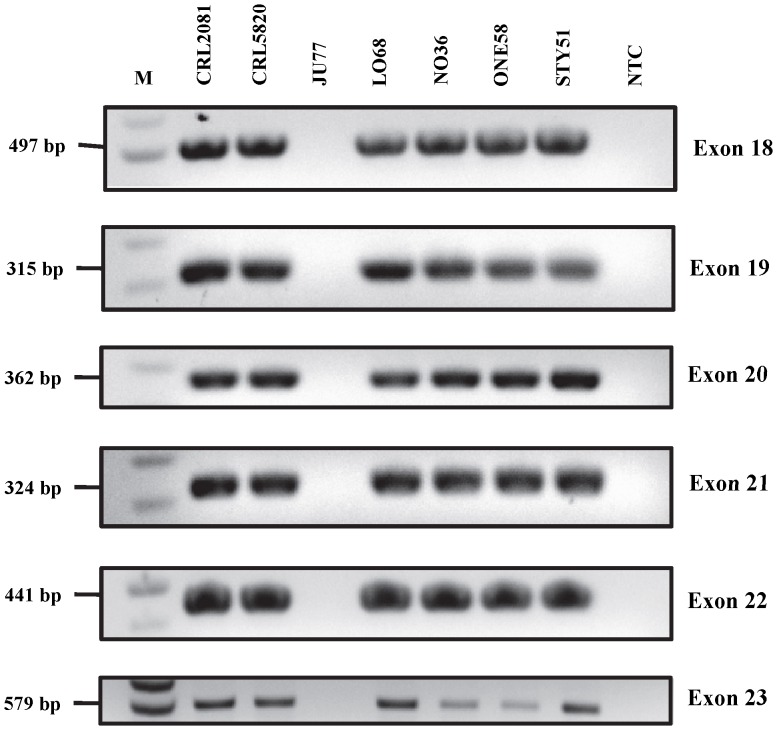
Agarose gel electrophoretic analysis of amplified exons 18–23 of *PTCH1* gene from MMe cell lines. JU77 has a homozygous deletion of all 6 exons. Lanes are labeled according to the cell lines, “NTC” = No template control. Exons assayed and size markers (“M”), in base pairs, are shown on left side of the gel image.

**Figure 5:**
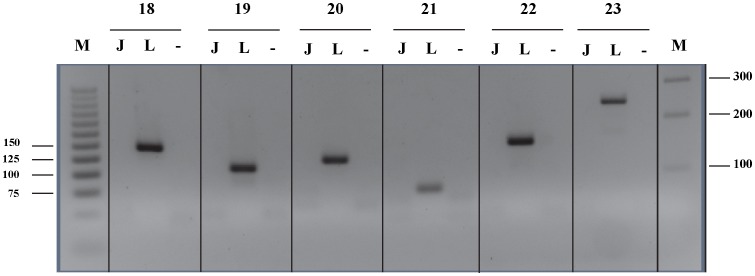
Confirmatory PCR. JU77 showing a deletion of exons 18–23 of the *PTCH1* gene. Lanes are labeled according to the cell lines (“L” = LO68, “J” = JU77 and “−” = NTC) and exons assayed and size markers (“M”), in base pairs, shown on both sides of the gel image.

**Figure 6:**
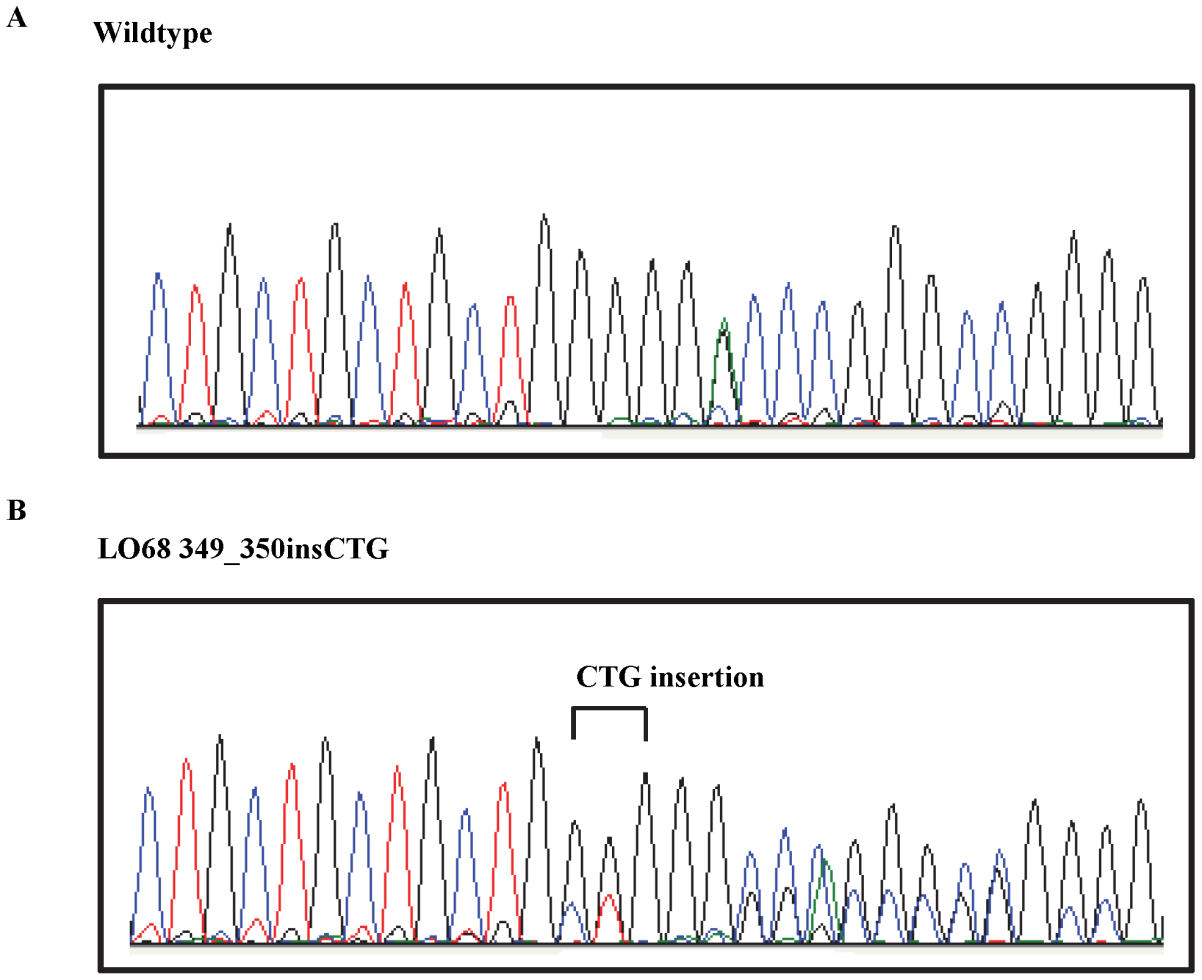
Identification of a *SMO* ^23insL^ mutation in LO68 cell line. **A**, Wild-type sequence in MSTO-211H cell line. **B**, 3-bp CTG insertion in LO68 cell line.

Although no point mutations were identified in the exonic region, homozygous deletion of exons 18, 19, 20, 21, 22 and 23 of *PTCH1* in JU77 cell line was observed and had been suspected by the absence of PCR product on agarose gel electrophoresis (Figure 4). Subsequently, this was confirmed by PCR using exonic primers and demonstrated complete absence of PCR amplicon in the cell line (Figure 5). In addition to the identification and characterization of point mutations and exon deletions, a 3 base-pair insertion of CTG between nucleotides 69 and 70 in exon 1 of the *SMO* gene in LO68 cell line was identified. This 69_70insCTG mutation resulted in an in-frame addition of a leucine residue after amino acid 23 of the SMO protein (p.23L_24GinsL) with unknown functional role (Figure 6). The same in-frame insertion was recently reported in two out of 39 gastric tumors [56].

### Hedgehog Pathway Gene Variants in MMe Cell Lines

Screening all the exons of each of the 13 HH pathway genes revealed a total of 35 SNPs using our panel of 7 MMe cell lines (Table 4). Examination of the NCBI dbSNP database revealed that all the SNPs were previously reported variations. No polymorphism was found in the exonic regions of *DHH and SHH* genes. Among the 35 SNPs detected, all were located in the coding regions and 16 SNPs would cause substitution of an amino acid (Table 4). The most frequent SNPs include: (i) *GLI2* rs2592595, ii) *GLI2* rs3738880, iii) *GLI2* rs10167980, iv) *GLI2* rs12711538, v) *KIF7* rs3803531, vi) *KIF7* rs8037349, vii) *KIF7* rs8179066 and *SMO* rs2228617, which were identified in all 7 cell lines. *In silico* analysis of potential functional impact of the 16 non-synonymous SNPs were characterized by the SIFT and PolyPhen2 programs. There was an overlap between SIFT and PolyPhen2 predictions: only one SNP (*KIF7* rs8179065) was predicted to be a non-deleterious substitution by both SIFT and PolyPhen2 (Table 4). The remaining five non-synonymous SNPs (*PTCH1* rs357564, *STK36* rs1344642 and rs1863704, *KIF7* rs138354681 and *GLI1* rs2228224) were predicted by PolyPhen2 as damaging but benign by SIFT (Table 4).

**Table 4:** SNPs identified in MMe cell lines.

Gene	Exon	Region	Nucleotide change	Amino acid change	dbSNP ID	SIFT	PolyPhen2	Cell line
*GLI1*	5	Coding	576 G>A	E192E	rs2228225	Tolerated	Benign	JU77, LO68, NO36, STY51
*GLI1*	11	Coding	2798 G>A	G933D	rs2228224	Tolerated	Damaging	JU77, LO68, NO36, STY51
*GLI1*	11	Coding	3298 G>C	E1100Q	rs2228226	Tolerated	Benign	JU77, LO68, NO36, STY51
*GLI2*	5	Coding	801 G>A	S267S	rs2592595	Tolerated	Benign	MSTO-211H, NCI-H28, JU77, LO68, NO36, ONE58, STY51
*GLI2*	13	Coding	3466 G>T	A1156S	rs3738880	Tolerated	Benign	MSTO-211H, NCI-H28, JU77, LO68, NO36, ONE58, STY51
*GLI2*	13	Coding	3916 G>A	D1306N	rs12711538	Tolerated	Benign	MSTO-211H, NCI-H28, JU77, LO68, NO36, ONE58, STY51
*GLI2*	13	Coding	3939 A>G	P1313P	rs10167980	Tolerated	Benign	MSTO-211H, NCI-H28, JU77, LO68, NO36, ONE58, STY51
*GLI3*	4	Coding	547 A>G	T183A	rs846266	Tolerated	Benign	MSTO-211H, NCI-H28, LO68, ONE58, STY51
*GLI3*	14	Coding	2993 C>T	P998L	rs929387	Tolerated	Benign	JU77, NO36, ONE58
*HHIP*	13	Coding	2058 T>C	I686I	rs11727676	Tolerated	Benign	MSTO-211H, NCI-H28
*IHH*	3	Coding	600 G>A	T200T	rs3731878	Tolerated	Benign	STY51
*IHH*	3	Coding	753 T>C	P251P	rs3731881	Tolerated	Benign	MSTO-211H, NCI-H28, JU77, LO68, ONE58
*IHH*	3	Coding	1128 T>C	T376T	rs394452	Tolerated	Benign	MSTO-211H, NCI-H28, JU77, LO68, ONE58, STY51
*KIF7*	1	Coding	154 G>A	D52N	rs8179065	Tolerated	Benign	MSTO-211H, NCI-H28, LO68
*KIF7*	1	Coding	195 G>C	A65A	rs8179066	Tolerated	Benign	MSTO-211H, NCI-H28, JU77, LO68, NO36, ONE58, STY51
*KIF7*	4	Coding	1102 A>G	T368A	rs8037349	Tolerated	Benign	MSTO-211H, NCI-H28, JU77, LO68, NO36, ONE58, STY51
*KIF7*	11	Coding	2501 A>G	Q834R	rs138354681	Tolerated	Damaging	ONE58
*KIF7*	12	Coding	2658 A>C	A886A	rs3803531	Tolerated	Benign	MSTO-211H, NCI-H28, JU77, LO68, NO36, ONE58, STY51
*KIF7*	13	Coding	2873 G>T	S958I	rs3803530	Tolerated	Benign	
*KIF7*	14	Coding	3013 G>A	G1005R	rs12900805	Tolerated	Benign	JU77, ONE58, STY51
*KIF7*	14	Coding	3048 G>A	S1016S	rs9672286	Tolerated	Benign	JU77, ONE58, STY51
*PTCH1*	23	Coding	3944 C>T	P1315L	rs357564	Tolerated	Damaging	MSTO-211H, NCI-H28, ONE58, STY51
*PTCH2*	2	Coding	90 G>A	L30L	rs45573433	Tolerated	Benign	MSTO-211H, NCI-H28
*PTCH2*	8	Coding	1080 G>T	V360V	rs11573579	Tolerated	Benign	LO68
*PTCH2*	14	Coding	1821 A>G	E607E	rs2295997	Tolerated	Benign	NO36
*PTCH2*	14	Coding	2055 T>C	A685A	rs7525308	Tolerated	Benign	JU77, NO36
*PTCH2*	16	Coding	2487 C>T	D829D	rs2295996	Tolerated	Benign	JU77
*SMO*	1	Coding	74 A>G	D25G	rs41304185	Tolerated	Unknown	MSTO-211H, NCI-H28, LO68, NO36
*SMO*	2	Coding	384 C>T	A128A	rs45571737	Tolerated	Benign	MSTO-211H
*SMO*	4	Coding	808 G>A	V270I	rs111694017	Tolerated	Benign	LO68
*SMO*	4	Coding	852 G>A	Q284Q	rs45445295	Tolerated	Benign	ONE58
*SMO*	6	Coding	1164 G>C	G388G	rs2228617	Tolerated	Benign	MSTO-211H, NCI-H28, JU77, LO68, NO36, ONE58, STY51
*STK36*	13	Coding	1748 G>A	R583Q	rs1344642	Tolerated	Damaging	JU77
*STK36*	24	Coding	3008 G>A	G1003D	rs1863704	Tolerated	Damaging	JU77
*SUFU*	11	Coding	1299 T>C	I433I	rs17114803	Tolerated	Benign	NO36

### PTCH1, SMO and SUFU Mutations in FFPE MMe Tumors

To validate the mutations that were identified in the cell lines, we analyzed DNA from micro-dissected FFPE tumors from 14 patients with MMe for *PTCH1* exonic deletions, *SMO* insertion and *SUFU* point mutation. Sequencing results showed that one patient had a tumor harboring the CTG insertion in the *SMO* gene. The patient was a male Caucasian who survived for 3.4 months after being diagnosed at age 51 years with biphasic MMe. As blood was not collected from the patient, the germline vs. somatic origin of the mutation could not be determined. Lastly, we did not detect the presence of the PTCH1 exon deletions or the *SUFU* mutation in the patient cohort.

### Functional Characterization of SUFU Mutant

**Figure 7:**
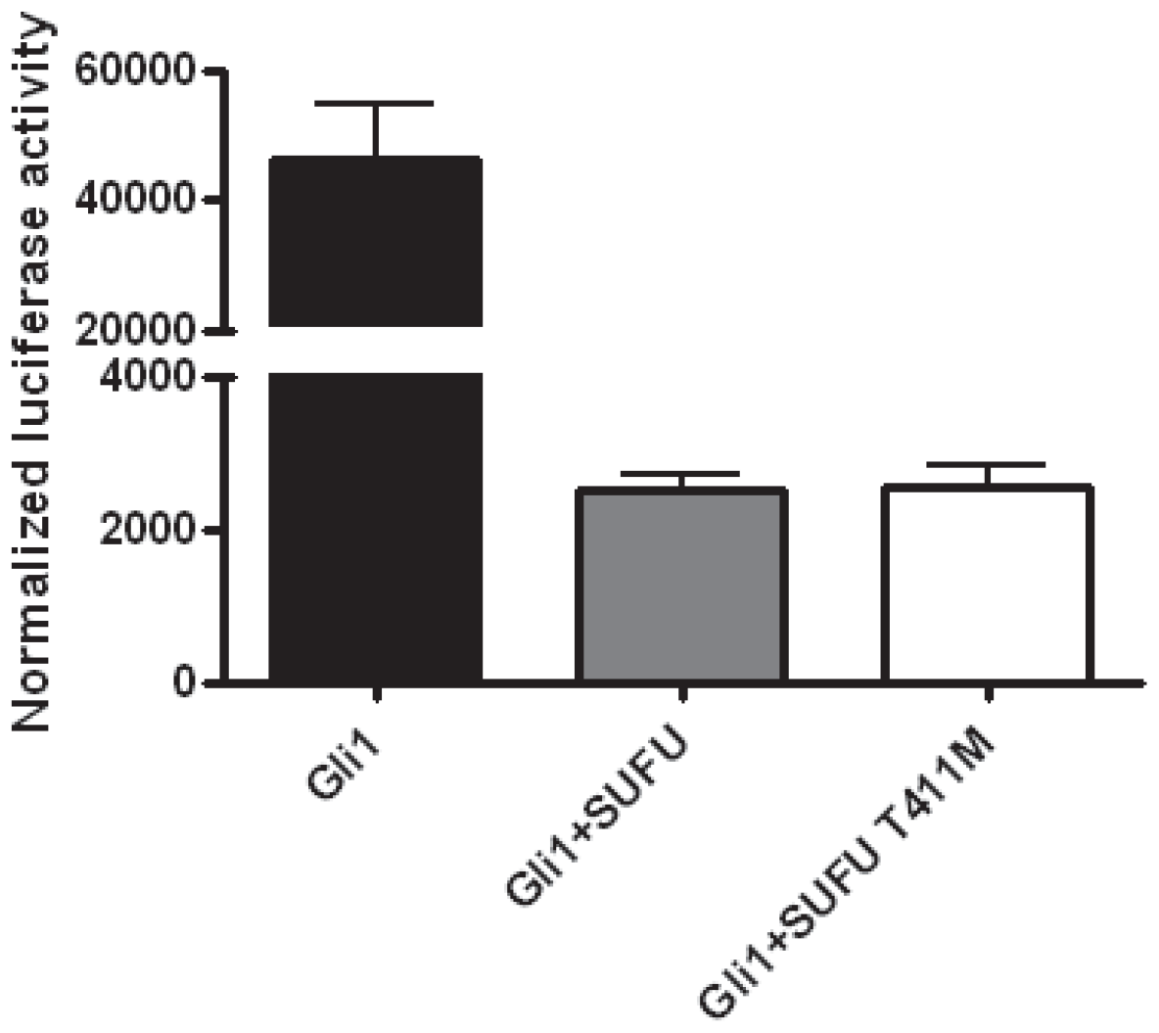
p.T411M mutation fails to disrupt the inhibitory function of SUFU. C3H10T1/2 cells were transfected with Gli-luciferase reporter construct with Renilla luciferase plasmid for 48 h before they were harvested for luciferase activity determination. Values represent the mean ± S.E.M. of a representative experiment performed in triplicates.

To corroborate the SIFT and PolyPhen2 predictions that the p.T411M mutation affects SUFU function, we transiently transfected C3H10T1/2 cells with a Gli1 luciferase reporter construct and expression constructs for wild-type Gli1, wild-type SUFU and the SUFU p.T411M mutant. Transfection of wild-type Gli1 resulted in increased Gli1 reporter activity whereas cotransfection of wild-type SUFU inhibited Gli1-induced reporter activity. However, the SUFU mutant failed to suppress Gli1 reporter activity (Figure 7).

## Discussion

We examined the expression profiles of the HH gene components in MMe cell lines and cultured primary mesothelial cells. We found that the HH pathway components *GLI1*, *GLI2* and *PTCH1* are expressed at high levels in MMe cell lines compared to normal mesothelial cells. This suggests that HH signaling is active in MMe, which corroborated findings from an earlier study [54], [55].

Aberrant activation of HH signaling in human cancers could result from genetic alterations in pathway components, including *PTCH1*, *SMO*, *SUFU* and *GLI1* [37], [42], [57], [58]. Utilizing *de novo* sequencing of PCR-amplified DNA fragments, we present for the first time a mutational analysis of 13 genes encoding components of the HH signaling pathway in 7 MMe cell lines. The rationale for screening HH pathway genes for genetic alterations is based on several observations: 1) this pathway plays a critical role in development and growth, 2) high frequency of mutations in the HH pathway genes screened to date, and 3) the HH pathway components were found to be overexpressed in primary MMe tumors and cell lines [54], [55].

In the present study, only three mutations were found in 3 out of 13 genes screened and they were detected in only two cell lines (Table 3). The first mutation that we have identified is a novel multiple-exon deletion in the *PTCH1* gene in JU77 cell line, whereby exons 18–23 were deleted. This mutation would result in a putative truncated PTCH1 protein, in which one of two extracellular loops and the cytoplasmic C-terminal domain are lost, or could undergo nonsense-mediated mRNA decay. However, we did not detect this multi-exonic deletion in our cohort of tumor samples from patients with MMe, but this may be due to the small sample size of the patient cohort.

The tumor suppressor gene *PTCH1* was cloned in 1996 and subsequently shown to be involved in repression of the HH pathway [13], [21], [22]. As expected for a classical tumor suppressor gene, *PTCH1* was found to be mutated in Gorlin syndrome and many other cancers [59]. Previously published studies on mutations in *PTCH1* have found more than 300 mutations, most of which appeared to be clustered in the large extracellular and intracellular loops of the PTCH1 protein [59], [60]. Although the functional impact of the deletion of exons 18–23 in *PTCH1* has yet to be elucidated, the key role of the extracellular loops in mediating HH ligand binding and the role of the cytoplasmic C-terminal domain in mediating subcellular localization and turnover of PTCH1 as well as inhibition of HH gene targets suggest that the receptor lacking exons 18–23 may lead to an aberrantly activated HH pathway [61]–[63]. Recent studies also suggest that the C-terminal fragment of PTCH1 could be localized to the nuclear region of the cell where it represses the transcriptional activity of Gli1, even though the canonical view of PTCH1 as a transmembrane protein is deeply entrenched in the literature [64]. In addition to the exonic deletion, we found a SNP (c.3944C>T) in exon 23 of *PTCH1*, which was also reported in cancers of the skin, bone and vulva [32], [65]–[68]. This non-synonymous SNP leads to substitution of leucine for proline at position 1315 in the C-terminal domain of the PTCH1. Modeling analysis suggested that the Leu^1315^ substitution might alter the secondary structure of PTCH1 resulting in constitutive pathway activation [69]. Intriguingly, this biallelic SNP has recently been associated with a higher risk for development of breast and nonmelanoma skin cancers [69]–[71]. The significance of this SNP with respect to etiopathogenesis of MMe, however, requires further study.

We also detected a *SMO* mutation in one MMe cell line which was also present in a tumor sample. SMO acts as a signal transducer of the HH pathway, mediating communication between transmembrane PTCH1 receptor and transcriptional activators GLI1 and GLI2. Xie *et al*. found activating somatic mutations in *SMO* in 3 out of 47 (6.4%) patients with sporadic BCC [37]. Xie *et al*. went on further to show that overexpression of these mutant SMO proteins in mouse skin produced BCC-like tumors [37]. In this study, the identified mutation, 23L_24GinsL, lies in the signal peptide region of SMO and has been detected in two cases of human gastric cancer {Wang, 2013 #657}. This mutation might affect the processing of the SMO precursor and in turn potentially interfere with protein targeting to the cell membrane.

One of the most intriguing results of this analysis is the identification of a mutation in *SUFU*. SUFU was originally identified as a negative regulator of the HH pathway in early embryonic development [72]. Taylor *et al.* first implicated SUFU in the tumorigenesis of childhood MB in 2002 [73]. They identified germline and somatic *SUFU* nonsense mutations in 8.7% (4 of 46) of desmoplastic MBs. Subsequently, *SUFU* mutations were found in 4.8% (2 of 42) and 2.5% (2 of 83) of sporadic BCCs and MBs, respectively [33], [74]. We found a missense mutation affecting Thr^411^ in SUFU in one of the 11 cell lines. Importantly, the same mutation was recently detected in a patient with colorectal cancer as part of the Cancer Genome Atlas [75], supporting a role of this mutation in tumorigenesis. This mutation was predicted by two web-based programs, SIFT and PolyPhen2, that predict the potential functional impact of altered amino acid sequences on encoded protein function, to result in a major change in amino acid class (from small, polar to large, hydrophobic) in the N-terminal region located close to the GLI1 binding domain, thereby implying a negative impact on protein function. SUFU acts as a classic tumor suppressor gene, with mutations leading to the inability of SUFU to transport GLI1 out of the nucleus to the cytoplasm, thereby resulting in aberrant activation of HH signaling in MB [73]. However, functional characterization showed that the p.T411M mutation does not alter the negative regulatory function of SUFU in a Gli1 luciferase reporter assay.

In addition to *PTCH1*, *SUFU* and *SMO*, we screened our MMe cell line panel for mutations in 9 other HH pathway genes *PTCH2*, *DHH*, *IHH*, *SHH*, *GLI1*, *GLI2*, *GLI3*, *KIF7*, *STK36* and *HHIP*. We found no evidence for mutations in any of these genes in our cell lines. However, we did find a number of previously reported SNPs, of which 16 were shown to result in non-synonymous codon substitution. To explore the functional significance of these SNPs, all those that are non-synonymous were analyzed by SIFT and PolyPhen2. There was considerable difference between the predictions from different algorithms: a *KIF7* SNP rs8179065 was predicted by SIFT to have altered protein function whereas the PolyPhen2 program predicted the *PTCH1* SNP rs357564, *STK36* SNPs rs1344642 and rs1863704, *KIF7* SNP rs138354681 and *GLI1* SNP rs2228224 to be deleterious amino acid substitution. Algorithmic differences aside, the real functional significance of these non-synonymous SNPs on HH signaling needs to be validated by biochemical studies.

Recently, SNPs in *SHH*, *GLI2* and *GLI3* genes have been reported to be associated with clinical outcome of trans-urethral resection and Bacillus Calmette-Guerin intravesical therapy for non-muscle-invasive bladder cancer patients (NMIBC) [76]. The *GLI2* SNP rs11685068 and *SHH* SNP rs1233560 demonstrated significant associations with recurrence in NMIBC patients who received trans-urethral resection. On the other hand, the NMIBC patients receiving Bacillus Calmette-Guerin treatment who carried the *GLI3* SNPs rs3801192 and rs6463089 were found to have a higher cancer recurrence rate and shorter recurrence-free survival time compared to those carrying the wildtype genotype. In a separate genome-wide association study, common variants in the *SHH*, *BTRC* and *HHIP* genes have been associated with the risk of pancreatic cancer [77]. These studies demonstrated a link between common genetic variations in the Hedgehog pathway and cancer risk, thus highlighting the importance of examining the association between SNPs in HH pathway genes and the risk of MMe.

Clearly, the frequency of mutations in key HH pathway genes in MMe is less than in BCC and MB where the mutations lead to hyperactivation of the pathway. However, the importance of these and other unidentified mutations in HH pathway genes in the pathogenesis of a subset of MMe tumors cannot be discounted and needs further investigation in a larger sample set.

In summary, we report mutations in SMO and SUFU and a novel multi-exonic deletion in *PTCH1* in MMe cell lines and tumors. Our data suggest that unlike BCC, MB and rhabdomyosarcoma in Gorlin syndrome, aberrant activation of HH signaling in MMe is unlikely to be driven by mutations in the majority of tumors but instead activated through autocrine signaling as suggested by Shi et al. [54] This pathway may represent a novel therapeutic target in MMe for the recently developed HH pathway inhibitors.

## Supporting Information

Table S1 **List of primers used for the PCR amplification and sequencing of Hedgehog pathway genes.**
(XLSX)

Table S2 **PCR conditions used in this study.**
(XLSX)

Table S3 **List of primers used for the detection of PTCH1 exon deletions.**
(XLSX)
